# Multi-centre parallel arm randomised controlled trial to assess the effectiveness and cost-effectiveness of a group-based cognitive behavioural approach to managing fatigue in people with multiple sclerosis

**DOI:** 10.1186/1471-2377-10-43

**Published:** 2010-06-16

**Authors:** Peter W Thomas, Sarah Thomas, Paula Kersten, Rosemary Jones, Alison Nock, Vicky Slingsby, Colin Green, Roger Baker, Kate Galvin, Charles Hillier

**Affiliations:** 1 Dorset Research and Development Support Unit, Poole Hospital NHS Foundation Trust, Poole, Dorset, UK; 2 The School of Health and Social Care, Bournemouth University, Bournemouth, Dorset, UK; 3 School of Health Sciences, University of Southampton, Hampshire, UK; 4 Joint Clinical Research Unit, BrAMS Building, University Hospitals and Frenchay Hospital Bristol, UK; 5 Dorset MS Service, Poole Hospital NHS Foundation Trust, Poole, Dorset, UK; 6 Peninsula Medical School, University of Exeter, Devon, UK; 7 Dorset Healthcare NHS Foundation Trust, Bournemouth, Dorset, UK

## Abstract

**Background:**

Fatigue is one of the most commonly reported and debilitating symptoms of multiple sclerosis (MS); approximately two-thirds of people with MS consider it to be one of their three most troubling symptoms. It may limit or prevent participation in everyday activities, work, leisure, and social pursuits, reduce psychological well-being and is one of the key precipitants of early retirement. Energy effectiveness approaches have been shown to be effective in reducing MS-fatigue, increasing self-efficacy and improving quality of life. Cognitive behavioural approaches have been found to be effective for managing fatigue in other conditions, such as chronic fatigue syndrome, and more recently, in MS. The aim of this pragmatic trial is to evaluate the clinical and cost-effectiveness of a recently developed group-based fatigue management intervention (that blends cognitive behavioural and energy effectiveness approaches) compared with current local practice.

**Methods/Design:**

This is a multi-centre parallel arm block-randomised controlled trial (RCT) of a six session group-based fatigue management intervention, delivered by health professionals, compared with current local practice. 180 consenting adults with a confirmed diagnosis of MS and significant fatigue levels, recruited via secondary/primary care or newsletters/websites, will be randomised to receive the fatigue management intervention or current local practice. An economic evaluation will be undertaken alongside the trial. Primary outcomes are fatigue severity, self-efficacy and disease-specific quality of life. Secondary outcomes include fatigue impact, general quality of life, mood, activity patterns, and cost-effectiveness. Outcomes in those receiving the fatigue management intervention will be measured 1 week prior to, and 1, 4, and 12 months after the intervention (and at equivalent times in those receiving current local practice). A qualitative component will examine what aspects of the fatigue management intervention participants found helpful/unhelpful and barriers to change.

**Discussion:**

This trial is the fourth stage of a research programme that has followed the Medical Research Council guidance for developing and evaluating complex interventions. What makes the intervention unique is that it blends cognitive behavioural and energy effectiveness approaches. A potential strength of the intervention is that it could be integrated into existing service delivery models as it has been designed to be delivered by staff already working with people with MS. Service users will be involved throughout this research.

**Trial registration:**

Current Controlled Trials ISRCTN76517470

## Background

Multiple sclerosis (MS) is a chronic, unpredictable, incurable, demyelinating disease of the central nervous system affecting approximately 2.5 million people [1-3]. It is more common in women and onset peaks between 20 and 40 years. The causes and early development of the disease are not fully understood but probably involve immune, genetic, and environmental factors [2,3]. Fatigue is one of the most commonly reported and disabling symptoms of MS, often occurring daily [4] and with a variable course [5,6]. Up to 86% of individuals with MS experience fatigue at any one time; 65% consider it to be one of their three most troubling symptoms [4].

Fatigue has been defined as a 'subjective lack of physical and/or mental energy that is perceived by the individual or caregiver to interfere with usual or desired activities', p. 2 [7]. The pathophysiology of fatigue is unclear [8] but likely to be multi-factorial [9,10]. Findings on the relationships between fatigue and other clinical variables (such as age, gender, disease duration, and clinical activity) have been equivocal [11].

Researchers have distinguished between primary and secondary fatigue [12]. 'Primary' fatigue relates to aspects of fatigue deemed to be directly related to the disease process such as lassitude or asthenia (an overwhelming sense of tiredness not directly related to participation in activity or exercise), 'short-circuiting' fatigue (when muscular performance deteriorates during sustained activity but recovers after a short rest break) and heat sensitive fatigue (where fatigue is triggered or worsened by heat).

'Secondary' fatigue refers to fatigue that is not unique to MS and is related to factors common to a range of chronic and disabling conditions (e.g. sleep disturbance, medication side effects, infection, physical exertion, depression, anxiety, stressful life events, characteristics of the local environment - such as lighting and temperature within a work setting). The relationship between these dimensions is complex; various symptoms of MS may act as predisposing factors for secondary fatigue.

Fatigue may limit or prevent participation in everyday activities, work, leisure and social pursuits, restrict role fulfilment and reduce psychological well-being [12,13] and is one of the key precipitants of early retirement [14,15]. Its 'invisible' nature may lead to difficulties in personal and work relationships [16,17].

Fatigue is highly related to an individual's sense of control over MS and psychological well-being [13,18,19]. Sense of control has been found to predict lower levels of fatigue [20], suggesting that increasing self-efficacy related to fatigue could improve quality of life.

Pharmacological and non-pharmacological treatments are available for MS-related fatigue, but evidence on effectiveness is mostly inconclusive or non-existent [4,21,22]. Non-pharmacological studies exploring the effectiveness of energy conservation programmes for MS-fatigue have tended to be small and uncontrolled [23-27]. Two fatigue management initiatives have been developed in the UK [28,29]. Only the former has been evaluated and numbers were small. In the USA, a randomised controlled trial (RCT) [30] of an energy conservation course [31] found evidence for its effectiveness in reducing fatigue impact, increasing self-efficacy and aspects of quality of life; benefits were maintained at 1 year [32]. Results from a German adaptation were also promising [33]. A RCT that evaluated a multi-disciplinary fatigue management programme [34] demonstrated no reduction in fatigue impact compared with a placebo intervention.

Although the important relationships between physical and psychological aspects of MS-fatigue are recognised [35], high quality RCTs of psychological interventions are rare [36]. In Chronic Fatigue Syndrome (CFS) individual Cognitive Behavioural Therapy (CBT) is effective in treating fatigue [37,38]. A systematic review [39] of psychological interventions for MS identified just one RCT that used a cognitive behavioural approach to manage MS-fatigue [40]. The intervention consisted of individual CBT conducted by a clinical psychologist and was shown to be effective in reducing fatigue. However, in the UK National Health Service (NHS), and elsewhere, psychologists working with people with MS are scarce, thus this approach may prove impractical [39].

A number of pilot studies have used group-based cognitive behavioural approaches, either in the context of coping with MS [41-43], or specifically with MS-fatigue [44]. Group-based approaches are potentially more cost effective than one-to-one and offer opportunities for peer support. Recently, we have developed a group-based manualised fatigue intervention for the management of MS-fatigue that incorporates energy effectiveness and cognitive behavioural approaches [45]. This intervention involves health professionals routinely involved in the management of MS, supported by clinical psychologists, and is thus compatible with a wide range of existing health service structures (such as the UK National Health Service (NHS)). Pilot work has been encouraging and this trial is a formal evaluation of the effectiveness of the intervention [45].

### Aims

Patient population: Adults with multiple sclerosis experiencing significant fatigue that is impacting on daily life.

#### Primary aim

1. To test whether those allocated to the group-based cognitive behavioural fatigue management intervention differ (in terms of fatigue severity, self-efficacy, and MS-specific quality of life) from those allocated to current local practice.

#### Secondary aims

2. To test whether those allocated to a group-based cognitive behavioural fatigue management intervention differ (in terms of fatigue impact, mood, general quality of life, and activity patterns) from those allocated to current local practice.

3. To assess how the group-based cognitive behavioural intervention and current local practice differ in terms of cost-effectiveness.

4. For those who attend the group-based programme, to gather feedback about experiences of attending the programme, any changes made and barriers to change encountered, and helpful or unhelpful aspects.

## Methods/Design

### Trial design

**Figure 1:**
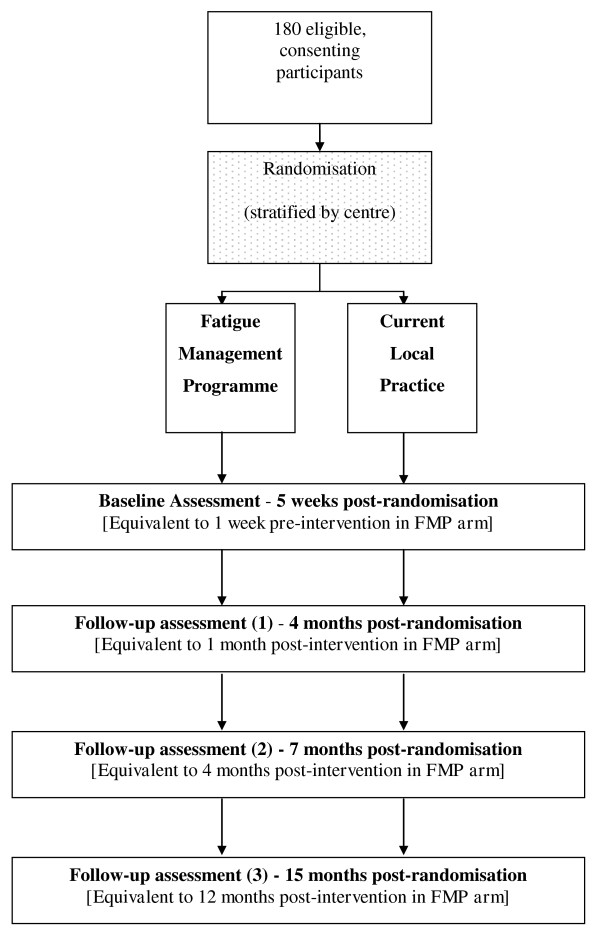
**Flowchart of trial design**.

This is a parallel arm randomised controlled trial comparing a group-based cognitive behavioural approach to managing fatigue (Fatigue Management Programme (FMP)) with current local practice. The trial design is summarised in Figure 1.

This RCT is the fourth stage of a research programme that has followed the Medical Research Council (MRC) guidance for developing and evaluating complex interventions [46]. The pilot work has been published [45].

Participants will be randomised to receive the fatigue management intervention or current local practice. A placebo arm to the trial was considered but rejected because (a) the fatigue management programme is a "complex intervention" (incorporating group, educational, cognitive, behavioural, and energy effectiveness components and the attendance of supportive others) making it difficult to know which "active ingredients" to control for in the placebo; (b) of the difficulties inherent in designing a placebo intervention that is credible to participants and facilitators, and that maintains participant masking, and (c) in clinical practice, the costs of delivering the fatigue management programme and the sham intervention would be equivalent.

The pilot research (multi-centre pilot) undertaken identified practical difficulties involved in running a three centre randomised trial. The multi-centre approach for the randomised trial will help to ensure sufficient numbers of participants are recruited and that results are generalisable outside Poole, where the intervention was developed.

### Setting

The trial is taking place in three centres (Poole, Bristol, Southampton/Portsmouth) and each centre has a team of trained facilitators to deliver the fatigue management programme.

### Ethical, governance and management considerations

This trial has been reviewed and given a favourable opinion by the North Somerset and South Bristol Multi-Centre Research Ethics Committee (ref: 08/H0106/2). Poole Hospital NHS Foundation Trust is acting as sponsor. The study is funded by a project grant awarded by the Multiple Sclerosis Society of Great Britain and Northern Ireland. The study is included in the National Institute of Health Research Clinical Research Network (NIHR CRN) portfolio (ID 4843).

As the trial involves people with MS and fatigue, careful consideration will be given to the location of the intervention (e.g. public transportation, car parking, walking distances), and the comfort of the participants (room temperature, refreshment breaks, cushioned chairs etc.). Because participants will have fatigue, the length of sessions in the fatigue management programme has been kept reasonably short, and the number and length of outcome measures have been kept to a minimum. Two facilitators will run each session. If someone becomes emotionally upset or unwell during a session, one of the facilitators can take that person aside, and provide information regarding help and support available from local MS services (such as a MS Specialist Nurse), the MS Society and MS Trust, and primary care (General Practice, counselling services etc.).

A trial steering group will meet at regular intervals throughout the trial.

### Service user support

There has been service user involvement in all stages of the research so far, including the development of this protocol. Service users will be represented on the trial steering group.

### Data protection

All information that is collected will be kept strictly confidential and any information that leaves Poole Hospital will not contain any personal details. Questionnaires will be allocated a participant identification number; they will not contain any names or identifying details. Only the authorised members of the research team will have access to the trial data.

### Participants

#### Sample size

Sample size consideration is mostly based upon fatigue as the primary outcome measure; this is the outcome measure used most frequently in other trials that include people with fatigue. As a variety of fatigue measures have been used in other trials, we have used standardised effect sizes to enable comparisons between them. Standardised effect sizes of 0.2 are commonly considered small, 0.5 considered medium, and 0.8 considered large [47]. In this trial, we will aim to detect a medium effect size. The Cochrane systematic review of cognitive behavioural therapy in people with CFS [38] identified two good quality studies, and in these the standardised effect sizes for fatigue were 0.7 and 1.0. The Cochrane Systematic Review of Amantadine for fatigue in MS found effect sizes of between 0.3 and 1.4 for the three studies that measured fatigue on a continuous scale [21]. Thus, our choice of an effect size seems reasonable in the context of (a) how well cognitive behavioural approaches work in another chronic disease where fatigue is a major symptom, and (b) how well another treatment for fatigue works in MS. For 85% power, the sample size requirement (using a two-tailed 5% significance level) is 73 people per arm of the trial; 146 in total. Inevitably, there will be some participants who withdraw from the trial during its course, or more generally, do not complete outcome measures. To allow for this, we will aim to enrol 180 participants; 90 in each arm of the trial. Thus, if 20% of participants do not provide data on the primary outcome measures, the study would maintain statistical power. This figure of 20% will be reviewed during the study and sample size adjusted up or down accordingly.

### Eligibility criteria

#### Inclusion criteria

1. Providing written informed consent.

2. Over the age of 18.

3. Clinical diagnosis of relapsing-remitting or progressive multiple sclerosis (Poser/McDonald criteria [48-50].

4. Score on the Fatigue Severity Scale (FSS) [51] greater than 4. The FSS is a uni-dimensional self-report measure consisting of 9 items that ask about severity of fatigue related to daily activities (such as physical functioning, exercise, work, family, and social life). Responses are made on a 7-point Likert-type scale.

5. Ambulatory (score on the Adapted Patient Determined Disease Steps (APDDS) Scale [52] < 8). This self-administered instrument is based on the Patient-Determined Disease Steps (PDDS) scale and the telephone Expanded Disability Status Scale (EDSS). The programme content will be most relevant for those who are ambulatory. Individuals who are non-ambulatory will continue to receive standard care.

6. Able to attend the intervention sessions.

7. English speaking.

#### Exclusion criteria

1. Attended a specific fatigue management programme within the last year.

2. Received a substantive, specific, fatigue intervention from an Occupational Therapist (OT) or other health professional, consisting of more than general advice, within the previous 3 months (such as the guidance produced by the National Association of Neurological Occupational Therapists (NANOT) [53] (now known as the College of Occupational Therapists Specialist Section - Neurological Practice) [29].

3. Already involved in another research study.

4. Individuals who have cognitive deficits such that they would not be able to engage in the group format or benefit from the programme. If individuals have substantial cognitive deficits much of the content of the fatigue management programme would not be appropriate. This will be based on the judgement of health professionals/local investigators.

5. Individuals who have had a relapse within the previous three months. This would be a potential confound since a change in fatigue could be the result of treatment or improvement after a recent relapse.

6. Individuals who have been on a disease-modifying drug (such as Beta-Interferon, Glatiramer Acetate) or an anti-depressant for fewer than 3 months. One of the possible initial side effects of these drugs is fatigue and thus this could be a confounder.

7. Individuals who are known to be currently under the care of a psychiatrist or under the care of addiction services will be excluded. Ongoing psychiatric disorders or addiction problems would compromise the potential to benefit from the programme.

Individuals excluded from the research project will continue to be seen as per usual care.

#### Source of participants

The recruitment target for each of the three centres will be 60 people, of whom 30 will receive the group-based intervention. In each centre this will require running three successive iterations of the programme, recruiting in blocks of around 20. The fatigue management programme has been designed for groups of between 6-12 participants.

Participants will be recruited using a variety of methods; via MS Services/Neurology Departments, MS Society newsletters and website, other relevant newsletters, MS Research database, MS support groups, General Practitioners.

#### Recruitment and consent

Potential participants (identified via secondary or primary care or self-referred) will be sent a trial information pack (Key Facts sheet, a set of Participant Information Sheets, two screening measures (FSS & APDDS), response slips (interested to hear more/decline to participate, plus reasons why) and a prepaid envelope. They will be asked to return a reply slip and the two completed screening measures in a prepaid envelope to the Local Investigator (LI) if they wish to find out more about the trial, or a decline slip, if they would prefer not to receive any further information. Individuals who have not responded will be sent a second trial information pack two weeks later and a final invitation to participate one month later.

The LI in each centre will telephone those who have expressed an interest in the trial and give them an opportunity to ask questions. If, after this discussion, the individual still wishes to take part, the LI will undertake a screening using a checklist. After screening, participants will be notified via the telephone whether they are eligible or ineligible. Participants who are currently ineligible (e.g. due to medication recently started, a planned holiday, having received fatigue advice etc.) but who may fulfil eligibility criteria at a later date will be held over to be rescreened (if another iteration is scheduled in the centre). Individuals who are ineligible will be sent a letter notifying them of this, along with a booklet produced by the MS Society about MS-fatigue [54].

#### Randomisation

To ensure good allocation concealment, random allocation will be e-mail-based and administered at Poole Hospital by the statistician who will be blinded to the identity of participants. Once a block of participants from a centre have provided informed consent they will be formally entered onto the trial database and an anonymised list of their identification numbers will be sent to the statistician who will randomly allocate half to the FMP group and half to the current local practice group. This method implies stratification by centre.

### Outcome Measures

The following demographic information will be collected before the start of the trial: age, sex, educational attainment, marital status, number of children, length of time since diagnosis, disease course, relapse history, employment status, prescribed drugs currently taken, co-morbid medical conditions, disability level.

For those randomised to the FMP, outcomes will be measured 1 week prior to the start of the programme and 1 month, 4 months and 12 months after the end of the programme. For those randomised to current local practice, outcomes will be sent on the same dates as those randomised to the FMP.

### Primary Outcome Measures

These are all self-reported questionnaire measures and specified *a priori*.

There are three primary outcomes:

#### 1. Self-reported fatigue severity

The Fatigue Assessment Instrument (FAI) [55] is an expanded version of the uni-dimensional Fatigue Severity Scale (FSS) [51]. The FSS is one of the best known and most used fatigue scales. It principally measures the impact of fatigue on specific types of functioning. The FAI has four subscales: fatigue severity, situation specificity, consequences of fatigue, and responsiveness to rest/sleep. Responses are made on a 7-point Likert-type scale. The fatigue severity subscale of the FAI corresponds almost exactly to the Fatigue Severity Scale, sharing eight of the original nine items along with three additional items. Scores on this subscale are a primary outcome. The other subscales are secondary outcomes.

#### 2. Self-reported MS-specific quality of life

The Multiple Sclerosis Impact Scale (MSIS-29) [56,57] measures the physical (20 items) and psychological impact (9 items) of MS on day-to-day life. It uses 5-point Likert-type scales ranging from 'not at all' to 'extremely' and is based on quality of life in the last two weeks. The total score for the MSIS-29 is a primary outcome measure. The physical and psychological subscales will be secondary outcomes.

#### 3. Self-reported self-efficacy for managing fatigue

The Multiple Sclerosis-Fatigue Self-Efficacy (MS-FSE) scale is adapted from the Control subscale of the MS Self-Efficacy (MSSE) Scale developed by Schwartz [58]. This adapted scale has undergone a preliminary validation in our pilot research.

### Secondary Outcome Measures

#### Self-reported fatigue

Three of the subscales from the FAI [55] (namely, situation specificity, consequences of fatigue and responsiveness to rest/sleep) along with the total score of the FAI.

#### Self-reported MS-specific quality of life

The subscale scores of the MSIS-29 [56,57] for the physical and psychological impact of MS.

#### Self-reported general quality of life

1. The Medical Outcomes Short-Form Survey version 2 (SF-36v2) [59,60] measures eight dimensions: physical functioning, role limitations because of physical health problems, bodily pain, general health perceptions, vitality, social functioning, role limitations because of emotional problems, and mental health. It generates scores for the eight dimensions as well as two summary measures (physical health and mental health). It uses Likert-type response scales. This measure will also be used to calculate Quality Adjusted Life Years (QALYs) for the economic analysis.

2. The EuroQoL (EQ-5D) [61] is a standardised instrument for use as a measure of health outcome. It has been developed by the EuroQoL Group as a simple, generic measure of health status, and is applicable to a wide range of health conditions. The EQ-5D consists of the EQ-5D descriptive system and the EQ visual analogue scale (EQ VAS) and it allows the derivation of a single index value for health status. The EQ-5D descriptive system comprises five dimensions (mobility, self-care, usual activities, pain/discomfort and anxiety/depression) each rated on three levels (no problems, some problems, severe problems). The EQ VAS records the respondent's self-rated health on a vertical, visual analogue scale where the endpoints are labelled 'best imaginable health state' and 'worst imaginable health state'.

#### Self-reported mood

The Hospital Anxiety and Depression Scale (HADS) [62] is a self-report measure consisting of an anxiety and a depression subscale. Each subscale consists of 7 items with a 4-point Likert-type response scale.

#### Self-reported fatigue severity

The Fatigue Symptom Inventory (FSI) [63,64] is a 14-item self-administered multi-dimensional questionnaire which measures the severity, frequency, and diurnal variation of fatigue and its perceived interference on quality of life. Severity is measured using four separate items that assess most, least, and average fatigue in the past week, as well as current fatigue. Frequency is measured using two separate items that assess the number of days in the past week that respondents felt fatigued, as well as the portion of the day on average they felt fatigued. Diurnal variation is measured using a single item that provides descriptive information about daily patterns of fatigue. Perceived interference is measured using seven separate items.

#### Self-reported sleep quality

These questions have been modified from the MS Clinical Practice Guidelines sleep questionnaire [7]. Questions include duration and quality of night-time sleep, factors that may prevent or interrupt sleep, and daytime sleeping and sleepiness.

#### Self-reported resource utilisation

A resource utilisation questionnaire will be administered at 4- and 12-month follow-up. It is adapted from one utilised in a large randomised controlled trial in Parkinson's disease [65], and includes questions about health and social service contacts in the preceding 3 months.

#### Self-reported fatigue management strategies

To gain deeper insights into the results of the trial, at the 4-month follow-up we will administer a semi-structured questionnaire to participants randomised to the FMP. In the questionnaire (which draws upon an existing questionnaire [66]) they will be asked to describe whether they have tried to make any changes to their lifestyle, behaviour, or thinking, as a result of the intervention; whether these changes have been made successfully or unsuccessfully, and the reasons why. The information they provide will be analysed both quantitatively (for example, number of lifestyle changes, number of successes) and qualitatively (identifying emergent themes in the responses given [67,68]). In this way, we will be able to look closely at "adherence" to the key principles of the intervention, which will help in the interpretation of the trial results.

#### Objective measure of physical activity

The *activ *PAL™ accelerometer classifies an individual's free-living activity into periods spent lying/sitting, standing and walking [69]. This information can be used to estimate daily energy expenditure, and time spent resting. Data will be collected at baseline and at 1- and 4-month follow-up. A postal method of administration was tested during an earlier research phase.

#### Self-reported satisfaction

Participants in the intervention arm will be asked to complete a brief semi-structured evaluation questionnaire at the end of each session of the fatigue management programme. This questionnaire was used in the pilot phases of the research [45].

### Intervention

The group-based fatigue management intervention [45] is based upon a conceptual framework that integrates elements from cognitive behavioural [70], social-cognitive [71], energy effectiveness, self-management [72] and self-efficacy [73] theories.

The intervention focuses on the management of fatigue, the most common symptom of MS, and is likely to be relevant to many people. However, it is expected that it will also provide a framework to help people manage their MS more generally. A cognitive behavioural approach that focuses on one symptom is likely to be clearer and less overwhelming than one encompassing many aspects, can be achieved in a smaller number of sessions, and will be easier to integrate within existing services.

The intervention consists of six sessions held on a weekly basis; the first, 2 hours' duration, and subsequent sessions, 1.75 hours' duration (each with a 15 minute refreshment break halfway through). It is designed to be run by two health professionals with experience of working with people with MS and of group-work (such as occupational therapists, nurses, or physiotherapists). This latter feature enables the intervention to be incorporated into existing health service structures. The intervention will be delivered to groups of 6-12 people.

Participants are encouraged to bring along a "supportive other" to the first session. Since the intervention entails participants making lifestyle changes, the involvement of a "supportive other" could help to encourage and support them in making such changes. Contact between members of the group outside the formal group setting will be encouraged, as an additional source of support.

The intervention is manualised and sessions are delivered via PowerPoint. A summary of programme content is presented in Table 1. The facilitator manual (~100 pages) provides detailed session information, guidance on preparation and delivery, a checklist of facilitator objectives and signposts to additional resources. There is also a companion participant workbook to reinforce material from the programme. Facilitators will be trained at one day orientation workshops and psychological advice and debriefing will be available for facilitators throughout the trial.

**Table 1:** Summary of content of the Fatigue Management Programme

Session	Title	Content	Homework
1	What is MS-related fatigue?	General introduction; expectations; icebreaker (quiz); types of fatigue; contributory factors, conceptual model of fatigue in MS	Activity/fatigue diary

2	Opening an "energy account"	Rest - functions; barriers; relaxation types and techniques; diaphragmatic breathing exercise; sleep hygiene	Rest/activity/sleep planner

3	Budgeting energy & smartening up goals	Types of activity; balancing activity & rest; moderating activity using the toolbox; goal-setting	Goal-setting exercise

4	The stress response; Introducing the cognitive behavioural model	The stress response (fight-or-flight); ways of coping with stress; introducing the cognitive behavioural model	'Unhelpful thoughts related to fatigue' diary

5	Putting unhelpful thoughts on trial	Unhelpful thought patterns related to fatigue; challenging unhelpful thoughts; levels of belief	Thought challenge sheet

6	Recapping & taking the programme forward	Revisiting expectations; group activity to revisit themes of the programme; rationale of 'Keeping on Track' planner	'Keeping on Track' planner

The aim of the intervention is to help people manage their fatigue by:

1. Normalising the experience of fatigue

2. Using their available energy more effectively

3. Developing "helpful thinking styles" about fatigue

#### Missed sessions

If participants do not attend a session they will be sent the materials they missed and, where possible, the session will be held over the telephone. Participants receiving the intervention will continue to have access to services available as part of their usual local care.

### Control group

Participants randomised to this arm of the trial will receive current local practice. Inevitably, there will be minor variations in the exact composition of what is usually provided, both within and between centres; depending on local resources and patient need. We consider this minor variation in current local practice to be a strength of the trial as it will increase the applicability of the findings to a wider range of centres. Individuals who have recently received substantive fatigue management are not eligible for the trial (see exclusion criteria).

## Results

### Statistical analysis

We will conduct two sets of statistical analyses. The primary analysis will be an intention-to-treat analysis, whereby participants are analysed in the arm of the trial they were randomised to, regardless of how many sessions of the programme they attend. In order for an intention-to-treat analysis to be conducted, we will endeavour to collect outcome measures for everyone (while recognising the right of participants to withdraw from the trial at any stage). The secondary analysis will be conducted on a "per protocol" basis. This analysis will exclude participants who attend fewer than four sessions of the fatigue management programme.

Data will be analysed primarily using SPSS for Windows. A 5% significance level will be used. Outcome measures will be assumed to be interval scaled, and the analysis will initially be focused on absolute change in outcome post-intervention relative to baseline, and absolute change in outcome at 4- and 12-month follow-up, relative to baseline. These change scores, presented separately for the three follow-up periods, are being used because we think they are the most clinically useful. They are likely to be normally distributed (this assumption will be checked). Change scores will be compared between the two groups using the independent samples *t*-test.

Additional analyses will be conducted to address the following issues:

1. We will adjust for baseline variability/baseline differences between treatment arms. For each outcome variable analysed, these would include the baseline measurement for that outcome, baseline primary outcome measures, gender, age, marital status, education level, type of MS, time since diagnosis, and level of disability.

2. Although participants in the trial have been randomised individually, for those randomised to the fatigue management intervention, it is possible that the group-based nature of the intervention will result in observations that are clustered. This could result in misleading statistical tests [74]. We will assess the extent to which this occurs by incorporating clustering into the analysis.

3. It is likely that outcome data will be missing for some participants. In the main analysis such individuals will be excluded. However, the implied assumption that the data are "Missing Completely At Random" might not be correct, potentially leading to biased estimates of the effect of the intervention [75]. Two methods will be used to assess the extent to which this occurs: Firstly, we will use the "Last Observation Carried Forward" imputation method (which assumes no change in outcome when a data point is missing). Secondly, we will use a mixed model for repeated measurements.

4. Analysing the change in outcome from baseline to each time point will, we believe, result in the most clinically useful presentation of results. However, to gain a deeper understanding of how outcome measures change over the course of follow-up, we will also model all measurement occasions together for each outcome.

5. It is possible that the fatigue management programme might be more effective in certain sub-groups of individuals. We will examine these potential effect modifiers by testing the relevant "treatment x effect modifier" interaction terms. However, we acknowledge that these statistical tests are likely to be underpowered, since this was not a specific aim of the study. In these analyses, we will consider study centre, baseline fatigue, gender, age, marital status, type of MS, time since diagnosis, and level of disability.

These additional analyses will involve the use of a variety of techniques including multi-level/mixed models. In addition to these pre-specified analyses, we will also conduct further exploratory analyses as suggested by the data.

Presentation of the results will follow the Consolidated Standards of Reporting Trials (CONSORT) [76,77] guidelines, in such a way that they can be meaningfully incorporated into systematic reviews. The design of the trial will help ensure that it is graded as high quality in systematic reviews.

### Economics evaluation

The economic burden of MS on the health services, on people with MS, their families (and/or carers) and society is high [78]. We recognise this as an important issue, and one that should be considered in the context of clinical trials. This is especially relevant in the United Kingdom NHS, where health policy decision makers (e.g. National Institute for Health and Clinical Excellence (NICE)) are interested in both the clinical value and cost-effectiveness of interventions. Therefore, alongside the proposed clinical trial, an economic evaluation will be undertaken to assess the incremental costs, incremental benefits, and the resulting cost-effectiveness of introducing a group-based cognitive behavioural approach to managing fatigue in people with MS, compared with current local practice (excluding the intervention).

It is anticipated that the primary economic endpoint will involve a clinically significant improvement in fatigue (e.g. cost per unit change in the fatigue outcome measure). Thereafter, the economic evaluation will estimate the cost per unit change in quality of life (e.g. MSIS-29, SF-36), and it will estimate the cost per Quality Adjusted Life Year (QALY) associated with the introduction of the group-based cognitive behavioural approach, as a more meaningful and policy relevant outcome. QALY estimates will be based on individual patient level data collected in the trial using the SF-36 and the EQ-5D. The economic assessment will explore the longer term consequences of improvements identified (expected) in fatigue and quality of life, modelling outcomes over time, where possible and appropriate, on the basis of the findings from the proposed trial. Where modelling of costs and consequences is undertaken, it will follow guidelines for good practice reported by Phillips and colleagues [79].

#### Perspective

A broad perspective to the measurement of costs and outcomes will be adopted, and results will be presented separately from the perspective of the NHS and personal social services (i.e. Third Party Payer), and from a broader societal perspective.

#### Data Collection

Trial data will be used to consider the relative effectiveness of the intervention versus comparator, and primary and secondary outcome measures have been described above. Patient level data will be collected within the trial both pre- and post-intervention, and at 4- and 12-month follow-up for those in the fatigue management arm of the trial and equivalent time points post-randomisation for those receiving current local practice. The primary economic analysis of outcomes will be between baseline and 4-month follow-up, although analysis using the shorter time horizon, and other longer term modelled outcomes will also be presented.

Resource use data will be collected over the 4-month follow-up period and will primarily comprise the direct delivery of the group-based cognitive behavioural approach (e.g. staff time, related consumables, and any travel costs for the health professional). Record forms will be used by staff delivering the intervention to identify staff time used, and the main categories of staff time (e.g. delivery of sessions, preparation, setting-up) and semi-structured interviews will be held with each of the Service Providers (centres) to estimate the broader resource use associated with delivery of the intervention.

Further data on NHS and personal social services resource use (e.g. related hospitalisation, Accident and Emergency Department visits, and General Practitioner visits) and information on carer time will be gathered using a simple patient self-completion questionnaire (completed at 4- and 12- month follow-up), and differences between groups will be investigated and reported as part of the economic analysis.

#### Cost Analysis

Cost analysis will estimate direct costs (i.e. those costs directly associated with the delivery of the intervention and the related follow-up of patients), using resource use data collected within the RCT, and staff and unit cost data from credible sources (national statistics and data from participating centres e.g. salary scales). Other costs will be determined using the data from the patient questionnaire and appropriate sources for unit cost data (e.g. Personal Social Services Research Unit (PSSRU), University of Kent at Canterbury, NHS Reference costs, local NHS Trust cost data). A mean net cost per patient in each arm of the trial and an incremental cost per patient, with associated measures of variance, will be calculated. Statistical analysis will characterise any uncertainty, and subgroup analysis (e.g. by disease severity) will be presented. All sources of cost data will be specified and all estimates will be transparent. Sensitivity analyses will be undertaken to address uncertainty within the unit cost estimates.

Where costs and benefits are considered beyond a 12-month time period, the evaluation will follow the present National Health Service (NHS)/Department of Health convention and discount future costs and future benefits at 3.5% per year. Analysis will also report non-discounted findings and findings where costs and benefits are subject to a range of discounted rates.

#### Sensitivity Analyses

Uncertainty in data estimates/assumptions will be subject to detailed sensitivity analysis, using plausible data ranges, where results indicate this to be appropriate (e.g. variations in staff grades and staff costs, variations in the estimates for NHS resource use and the relevant unit costs). Where data point estimates are not subject to sensitivity analysis, reasons for this will be given. Where undertaken, and as appropriate, sensitivity analysis will comprise one-way analysis, multi-way analysis, scenario analysis, and where modelling is undertaken, it will include probabilistic sensitivity analysis.

### Serious adverse events

Adverse events will be defined according to standard clinical trial definitions and serious adverse events will be reported to the Multi-centre Research Ethics Committee (MREC) within 15 days by the Chief Investigator.

### Data management

The trial data will be entered onto an SPSS spreadsheet by an administrator. A random 10% sample of the data will be checked for accuracy.

### Participant withdrawal of consent to research follow-up

If a participant withdraws consent to be included in research follow-up during the trial, the local investigator (LI) will be informed and will contact the participant. Provided the participant is willing to give a reason, the LI will find out why (s)he wishes to withdraw from the research follow-up. The LI will also determine whether the participant has given permission to retain data collected before withdrawal for use at final analysis, or whether this information should be destroyed. No data will be used in the analysis without a participant's consent.

## Discussion

This is a pragmatic trial aimed at answering the question of whether introducing the intervention into a service has a significant impact on fatigue, self-efficacy, and quality of life, and whether it represents value for money to the NHS. These are the most important questions from a health service and patient perspective.

A strength of this trial is the fact that the mixed methodology pilot work (intervention development and process and preliminary evaluation) underpinning the trial has been rigorously conducted and documented following the Medical Research Council guidance for the development of a complex intervention [46]. The intervention is fully manualised and supported by training and PowerPoint materials meaning that it can be easily replicated in a standardised form. The multi-centre nature of the trial gives the opportunity to test whether the intervention transfers successfully to other geographic areas. The intervention has been developed so that it can be delivered by those already routinely involved in the care of people with MS. Thus, if found to be beneficial, it could be readily incorporated into existing services, facilitating the integration of psychology-based approaches into patient care. The 12-month follow-up period offers the opportunity to explore and assess longer term effects.

Service users have been involved throughout the research process, from the pilot work through to the RCT (as lay grant application and document reviewers, focus group participants, trial participants and as members of the steering group). Participants randomised to the intervention arm will have an opportunity to provide satisfaction and process feedback via session evaluation questionnaires and a semi-structured questionnaire administered at the second follow-up.

## Competing interests

The authors declare that they have no competing interests.

## Authors' contributions

PT and ST were involved in the conception and design of the study. PT is the Chief Investigator and wrote the initial grant application and protocol. ST is the Trial Coordinator and had significant input into the trial design, initial grant application and protocol development/writing. ST and PT developed the semi-structured questionnaire for the qualitative component. PK and RJ are the Principal Investigators for the Southampton/Portsmouth and Bristol sites, respectively. They have advised on methodological issues and local aspects for the grant application and trial protocol. CH, AN & VS advised on clinical aspects related to the grant application/trial protocol. CG designed the economic evaluation, contributed to the grant application/protocol and has advised on general aspects of trial/study design. RB advised on psychological debriefing for facilitators. KG advised on the qualitative aspects of the trial. All authors read and approved the final manuscript.

## Pre-publication history

The pre-publication history for this paper can be accessed here:

http://www.biomedcentral.com/1471-2377/10/43/prepub
